# Alterations of gut microbiota composition in post-finasteride patients: a pilot study

**DOI:** 10.1007/s40618-020-01424-0

**Published:** 2020-09-19

**Authors:** F. Borgo, A. D. Macandog, S. Diviccaro, E. Falvo, S. Giatti, G. Cavaletti, R. C. Melcangi

**Affiliations:** 1 Department of Experimental Oncology, IEO European Institute of Oncology IRCCS, Milan, Italy; 2 Department of Oncology and Hemato-Oncology, Università Degli Studi di Milano, Milan, Italy; 3 Dipartimento di Scienze Farmacologiche e Biomolecolari, Università Degli Studi di Milano, Via Balzaretti 9, 20133 Milan, Italy; 4 Experimental Neurology Unit, Università di Milano-Bicocca, Monza, Italy; 5 Milan Center for Neuroscience, Università di Milano-Bicocca, Monza, Italy

**Keywords:** Androgenic alopecia, Fecal microbiota, Gut microbiota-brain axis, Sexual dysfunction, Depression, 5alpha-reductase

## Abstract

**Purpose:**

Post-finasteride syndrome (PFS) has been reported in a subset of patients treated with finasteride (an inhibitor of the enzyme 5alpha-reductase) for androgenetic alopecia. These patients showed, despite the suspension of the treatment, a variety of persistent symptoms, like sexual dysfunction and cognitive and psychological disorders, including depression. A growing body of literature highlights the relevance of the gut microbiota-brain axis in human health and disease. For instance, alterations in gut microbiota composition have been reported in patients with major depressive disorder. Therefore, we have here analyzed the gut microbiota composition in PFS patients in comparison with a healthy cohort.

**Methods:**

Fecal microbiota of 23 PFS patients was analyzed by 16S rRNA gene sequencing and compared with that reported in ten healthy male subjects.

**Results:**

Sexual dysfunction, psychological and cognitive complaints, muscular problems, and physical alterations symptoms were reported in more than half of the PFS patients at the moment of sample collection. The quality sequence check revealed a low library depth for two fecal samples. Therefore, the gut microbiota analyses were conducted on 21 patients. The α-diversity was significantly lower in PFS group, showing a reduction of richness and diversity of gut microbiota structure. Moreover, when visualizing β-diversity, a clustering effect was found in the gut microbiota of a subset of PFS subjects, which was also characterized by a reduction in *Faecalibacterium* spp. and *Ruminococcaceae* UCG-005, while *Alloprevotella* and *Odoribacter* spp were increased compared to healthy control.

**Conclusion:**

Gut microbiota population is altered in PFS patients, suggesting that it might represent a diagnostic marker and a possible therapeutic target for this syndrome.

## Introduction

The post-finasteride syndrome (PFS) is an emerging clinical problem observed in a subset of patients treated with finasteride for androgenetic alopecia. These patients, despite the suspension of treatment, reported a variety of persistent symptoms, including sexual dysfunction, psychological complaints, muscular problems, physical alterations, and cognitive complaints [1, 2]. Examples of them are feeling a lack of connection between the brain and penis, loss of libido and sexual drive, difficulty in achieving erection, genital numbness or paresthesia, depression, reduction in self-confidence, decreased initiative and difficulty in concentration, forgetfulness or loss of short-term memory, irritability, suicidal thoughts, anxiety, panic attacks, sleep problems, muscular stiffness and cramps, tremors, chronic fatigue, joint pain, and muscular ache [3–22].

Finasteride is an inhibitor of the 5alpha-reductase (5α-R). This enzyme represents a key step in the conversion of neuroactive steroids, such as progesterone (PROG) and testosterone (T) into their reduced metabolites, such as dihydroprogesterone (DHP), tetrahydroprogesterone (THP), and isopregnanolone in case of PROG and dihydrotestosterone (DHT), 5α-androstane-3α,17β-diol (3α-diol)  and 5α-androstane-3β,17β-diol (3β-diol) in case of T. These neuroactive metabolites exert an important physiological control of the nervous functions by activating classical and non-classical steroid receptors [23, 24]. In agreement, alterations of their levels have been reported in neurodegenerative and psychiatric disorders [25–27] as well as in PFS patients [14, 21, 22]. We have recently studied the PFS in a pre-clinical rat model, describing alterations of neuroactive steroid levels in brain areas [28] as well as depressive-like behavior coupled with cellular and molecular markers, such as a decrease in the neurogenesis and increased neuroinflammation and reactive gliosis, one month after drug treatment interruption [29]. Interestingly, the composition of the microbiota was altered in this experimental setup, with a decrease in *Ruminococcaceae* family and *Oscillospira* and *Lachnospira* genus [29]. There is a growing body of literature showing the relevance of the gut microbiota-brain axis both in human health and disease [30–32]. In particular, alterations in gut microbiota composition have been reported in patients with major depressive disorder [33–35] as well as in animal models of depression [36].

In this study, we analyze the composition of the fecal microbiota in PFS patients in comparison with a healthy cohort.

## Materials and methods

### Study design and sample preparation

PFS patients were recruited through the *Italian finasteride side effects network*. Twenty-three healthy men, aged 25–51 years who reported persistent sexual and mental health side effects after the use of 1–1.25 mg daily of finasteride (i.e., Propecia, Proscar, or generic finasteride) for androgenetic alopecia, were considered in the case group. Only subjects who had suspended finasteride treatment at least 3 months earlier, had not used drugs known to potentially interfere with microbiome analysis, and were not affected by psychiatric disorders or sexual dysfunction before finasteride use, were included. The study procedure was approved by the Ethics Committee of the University of Milano-Bicocca Monza-Italy, (protocol number 434/2018) and the participating subjects provided their written informed consent before enrollment. A questionnaire was used to evaluate both the absence of PFS signs and symptoms before the finasteride treatment and the presence of this accompanying signs and symptoms after the drug treatment. Although not validated, it represents the only available tool to systematically collect information on patient conditions and to assess the features of PFS. The same questionnaire was used in our previous observations on PFS patients [1, 2, 14, 21, 22]. The questionnaire was filled by the patient himself only after the description of the study design to the patient, in order to limit selection and recall bias. The patients answered to the following questions (Q) with never/sometimes/often/always: (Q1) Decreased self-confidence; (Q2) Decline of emotional verve, initiative, and desire to do; (Q3) Difficulty concentrating and focusing (brain fog); (Q4) Mental confusion; (Q5) Forgetfulness or loss of short-term memory; (Q6) Losing train of thought or reasoning; (Q7) Slurred speech or stumbling over words; (Q8) Irritability or easily flying into a rage; (Q9) Nervousness, agitation, and inner restlessness; (Q10) Depression, hopelessness, and feelings of worthlessness; (Q11) Suicidal thoughts; (Q12) Anxiety; (Q13) Panic attacks; (Q14) Sleep problems; (Q15) Loss of libido and sexual desire; Q16) Difficulty in achieving an erection; (Q17) Feeling of a lack of connection between the brain and the penis; (Q18) Genital numbness or paresthesia; (Q19) Feeling tinglings or pinpricks; (Q20) Tics and muscle spasms; (Q21) Tremors; (Q22) Involuntary muscle tension and contraction; (Q23) Dizziness; (Q24) Headache and migraine; (Q25) Chronic fatigue, weakness; (Q26) Joint pain and muscular ache; (Q27) Decreased body temperature; and (Q28) Photophobia and other visual problems.

All participants were Caucasian ethnicity adhering to Mediterranean diet and did not show any co-morbidities before and after finasteride treatment. No drug treatment was reported after finasteride treatment, including prebiotics, probiotics, and antibiotics. In addition, neither constipation nor Irritable Bowel Syndrome was registered.

The fecal gut microbiota composition in PFS patients was compared to healthy cohort. Stool samples were home collected and stored at − 80 °C until use. The raw sequences of the healthy cohort (ten male subjects) are available in Borgo and collaborators [37] under BioProject PRJNA401981. PFS patients and Healthy subjects were ethnicity, sex, diet, and BMI matched.

### Bacterial DNA extraction and 16S rRNA gene sequencing

Total bacterial DNA was extracted from 200 mg of stool samples using Dneasy Powersoil Pro Kit (Qiagen) following the manufacturer’s instruction. 16S rRNA gene amplicon libraries were performed with a two-step barcoding approach according to Diviccaro et al. [34].

Briefly, the 16S rRNA gene was initially amplified by interest-specific primers targeting V3 (5′-TCGTCGGCAGCGTCAGATGTGTATAAGAGACAGCCTACGGGNGGCWGCAG-3′) and V4 (5′-TCGTCGGCAGCGTCAGATGTGTATAAGAGACAGCCTACGGGNGGCWGCAG-3′) regions coupled with overhang adapters. The reaction was carried out in 25 µl volumes containing 6 ng/µl microbial DNA, 1 µM of each primer, and 2 × KAPA HiFi HotStart ReadyMix (Roche).

The following PCR program was used: initial denaturation at 95 °C for 3 min, followed by 25 cycles consisting of denaturation (95 °C for 30 s), annealing (55 °C for 30 s), and extension (72 °C for 30 s), and a final extension step at 72 °C for 5 min. PCR products were analyzed by 1% agarose gel electrophoresis for quantity and quality. Expected size of the products after Amplicon PCR step is about 550 bp.

DNA samples resulting from PCR step were amplified with dual-index primers using Nextera Xt Index Kit V2 Set A (Illumina). Library concentration and exact product size were measured using Agilent 2100 Bioanalyzer System (Agilent). A 20 nM pooled library and a PhiX control v3 (20 nM) (Illumina) were mixed with 0.2 N fresh NaOH to produce the final concentration at 10 pM each and injected on a Miseq Reagent Nano Kit V2 500 Cycles for obtaining a paired-end 2 × 150 bp sequencing. Sequencing was performed at the IEO Genomic Unit. Fastq files were checked for quality using FastQC (https://www.bioinformatics.babraham.ac.uk/projects/fastqc/) and data analysis was performed using QIIME2 suite. Denoising was performed using the deblur denoise-16S workflow, setting the trim length to 245 bp. Sequences from the PFS patients (*n* = 23) were analyzed together with a healthy dataset (*n* = 10) matched by sex (male) and BMI (normal), by merging the output feature table and representative sequences from deblur denoising step. Phylogenetic tree reconstruction was performed on the merged table and sequences by fragment insertion using SEPP, with the SILVA 128 database used as reference. Core diversity analysis was performed with the core-metrics-phylogenetic pipeline, using the output feature table from deblur and tree from SEPP, with the rarefaction depth set to 1336 reads. Taxonomic classification of sub-OTU sequences identified by deblur was performed using the sample-classifier pipeline, by training a Naive Bayes classifier on V3–V4-trimmed 16S sequences from the SILVA 132 database.

### Statistical analysis

Sample biodiversity (i.e., α-diversity) was evaluated according to different microbial diversity metrics including Chao1, Shannon index, Evenness, Observed OTUs, and Faith’s phylogenetic distance. Inter-sample diversity (i.e., β-diversity) was calculated by using both weighted and unweighted UniFrac and Bray–Curtis distance metrics. Principal Coordinates Analysis (PCoA) was performed using build-in functions in QIIME2. Groups significance, according to experimental design, was calculated by Kruskal–Wallis test for alpha metrics vectors, whereas PERMANOVA test for beta-metrics distance matrices. Statistical significance was calculated using a Mann–Whitney test for comparison within two groups or Kruskal -Wallis test with Dunn’s multiple comparison correction within more than two groups. P < 0.05 (*), P < 0.01 (**), and P < 0.001 (***) are regarded as statistically significant.

## Results

### General data of the PFS patients at the clinical evaluation

Twenty-three PFS men vs healthy cohort (ten male subjects) were evaluated. Table 1 reports anthropometric data (weight, height, and body mass index) and age at the time of enrollment. For PFS patients, the mean of treatment duration was 1176 days. The interval between finasteride withdrawal and clinical evaluation was very wide (range 433–7111 days, median 2465).

**Table 1:** Clinical data

Subjects	Age (years)	Height (m)	Weight (kg)	BMI (kg/m^2^)
Patient 1	51	1.72	64	21.63
Patient 2	25	1.8	83	25.62
Patient 3	36	1.62	48	18.29
Patient 4	41	1.7	68	23.53
Patient 5	45	1.79	68	21.22
Patient 6	34	1.7	65	22.49
Patient 7	36	1.8	77	23.77
Patient 8	44	1.83	74	22.10
Patient 9	51	1.83	80	23.89
Patient 10	25	1.93	77	20.67
Patient 11	30	1.87	90	25.74
Patient 12	37	1.76	62	20.02
Patient 13	36	1.9	80	22.16
Patient 14	28	1.83	81	24.19
Patient 15	38	1.82	78	23.55
Patient 16	43	1.71	60	20.52
Patient 17	43	1.78	80	25.25
Patient 18	42	1.84	76	22.45
Patient 19	33	1.74	75	24.77
Patient 20	32	1.85	78	22.79
Patient 21	30	1.78	77	24.30
Patient 22	50	1.72	83	28.06
Patient 23	37	1.92	88	23.87
Healthy 1	35	1.80	80	24.69
Healthy 2	38	1.71	65	22.23
Healthy 3	42	1.70	70	24.22
Healthy 4	38	1.83	74	22.10
Healthy 5	58	1.76	70	22.60
Healthy 6	51	1.78	65	20.52
Healthy 7	56	1.70	74.5	25.78
Healthy 8	60	1.83	65	19.41
Healthy 9	46	1.75	76	24.82
Healthy 10	63	1.76	73	23.57

No statistical significant differences vs controls were found for height (*p* = 0.25), weight (*p* = 0.34), and BMI (*p* = 0.91) with the exception of age (*p* = 0.002). However, as well reported in literature, the gut microbiota composition is only affected during infancy and at old ages, while it is stable during the adult age [38]. Therefore, we have considered these patients for the present study.

Frequency of the symptoms pre-finasteride and post-finasteride was self-reported by the patients filling a questionnaire at the moment of the gut microbiota evaluations. More of the 50% of PFS patients considered in our study never reported symptoms related with sexual dysfunction, psychological complaints, muscular problems, physical alterations, and cognitive complaints before finasteride treatment. The only exceptions were represented by irritability/easily flying into a rage (Q8) or anxiety (Q12), which were sometimes/often (69.6% and 65.2%, respectively) reported also before treatment. Most of the symptoms were sometimes/often/always reported by more of the 50% of PFS patients at the moment of the gut microbiota evaluations. Panic attacks (Q13), feeling tinglings/pinpricks (Q19), tremors (Q21), dizziness (Q23), or headache (Q24) were never reported by the majority of the PFS patients, instead (Q13 and 24: 52.2%; Q19 and 21: 56.5%; Q23: 69.6%).

### Gut microbiota structure in PFS patients

**Fig. 1:**
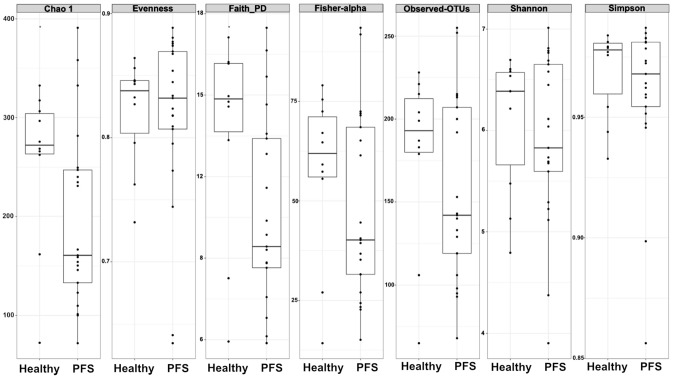
Alpha-diversity analysis in 10 healthy and 21 PFS patients. Intra-samples diversity of Healthy and PFS microbiota according to Chao 1 (*p* = 0.047), Evenness (*p* = 0.89), Faith PD, (*p* = 0.047), Observed-OTUs (*p* = 0.40), Shannon (*p* = 0.75)

The microbiota community structure of PFS subjects was examined in comparison to healthy cohort by α-diversity (within-sample diversity) metrics. Two PFS patients (i.e., the number 9 and 18 in Table 1) were characterized by a very low NGS library depth; therefore, we decided to remove them from gut microbiota evaluation. Significant differences among community richness were observed, as estimated by Chao1 (*p* = 0.047) index, showing a different gut microbiota structure in PFS subjects (Fig. 1). Phylogenetic diversity (Faith’s PD), which takes into consideration the phylogeny of microbes to estimate diversity across a tree, indicates a different richness and evenness (*p* = 0.047) (Fig. 1). No significant differences in diversity were observed between PFS and healthy subjects (Observed-OTUs, *p* = 0.40; Shannon, *p* = 0.75; Simpson, *p* = 0.89) (Fig. 1).

**Fig. 2:**
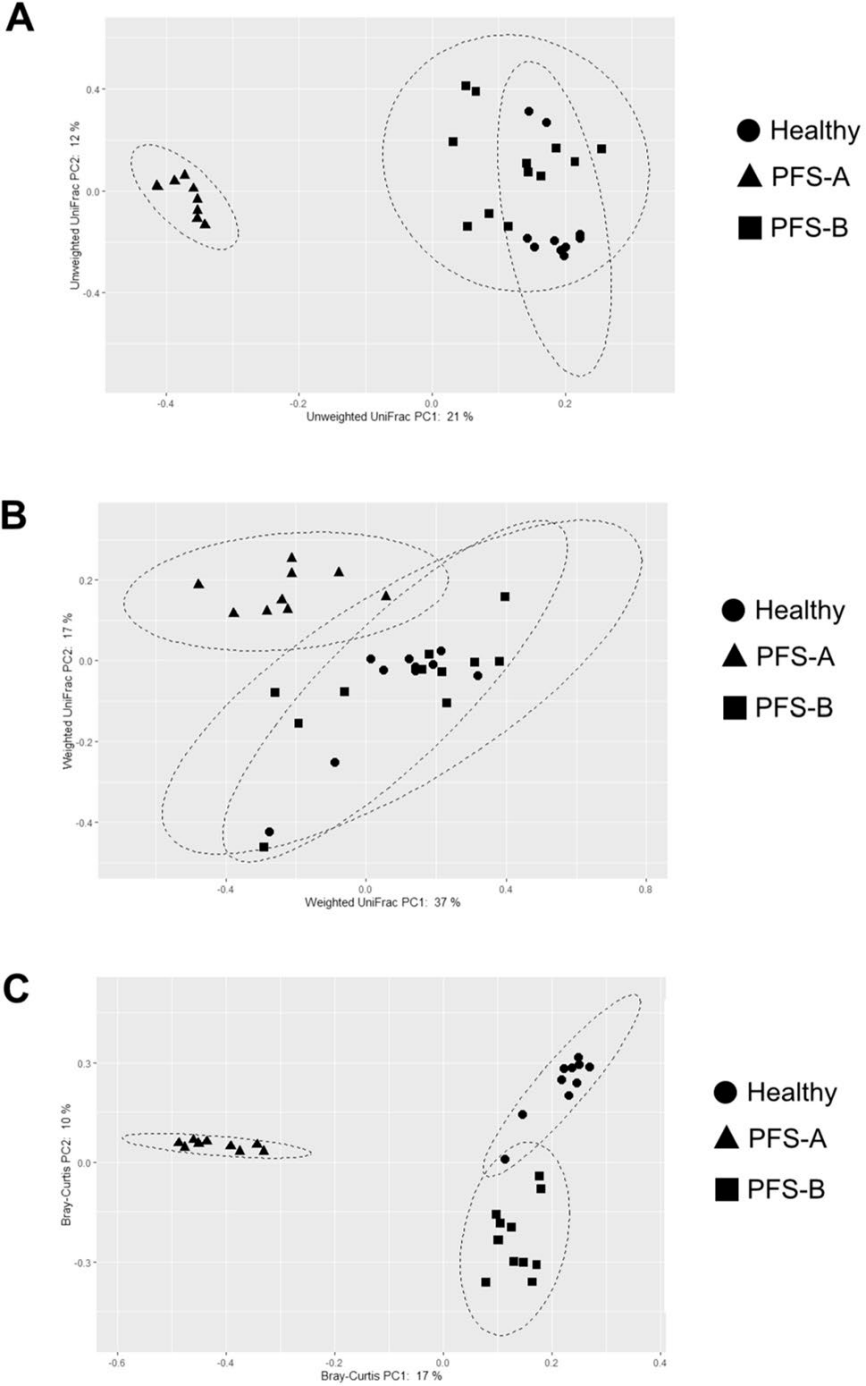
Beta-diversity analysis revealed two different sub-clusters in 21 PFS subjects. Unweighted UniFrac distance (**a**) showed significant separation for PFS-A and healthy subjects (*p* = 0.001), between PFS-B and healthy cohorts (*p* = 0.001), and within PFS subjects (PFS-A *vs* PFS-B; *p* = 0.001). Weighted UniFrac distance (**b**) showed a significant separation for PFS-A and healthy subjects (*p* = 0.001), and for PFS-A and PFS-B (*p* = 0.001), but no significant separation between PFS-B (*p* = 0.37) and healthy cohorts was detected. Bray–Curtis dissimilarity (**c**) showed a significant clustering (*p* = 0.001) for healthy and PFS sub-clusters

For gaining a more comprehensive view of diversity, we applied three different ß-diversity (between sample diversity comparisons) metrics visualized by Principal Coordinate Analysis (PCoA) (Fig. 2). PCoA is plotted for representing the microbial community compositional differences between PFS and healthy cohorts. The bacterial composition of these gut microbiota communities clustered according to the grouping. Unweighted UniFrac distance showed a significant clustering based on differences in low-abundance features (*p* = 0.001). The same clustering is partially confirmed by the weighted UniFrac distance (*p* = 0.05), underlining the significant differences in terms of microbial composition. In addition, we calculated the Bray–Curtis dissimilarity within PFS and healthy to quantify community similarity of the same sample type (*p* = 0.001). Community dissimilarity between samples within each group was smaller than dissimilarity between samples from different groups.

Interestingly, the analysis of the ß-diversity distinguishes two different sub-clusters within PFS patients (Fig. 2). In particular, the discrimination between PFS patients and healthy groups is driven by the sub-cluster A (Unweighted UniFrac, *p* = 0.001; Weighted UniFrac, *p* = 0.001; Bray–Curtis, *p* = 0.001), rather than the sub-cluster B (Unweighted UniFrac, *p* = 0.001; Weighted UniFrac, *p* = 0.37, Bray–Curtis metrics, *p* = 0.001) (Fig. 2). A strong separation was found for PFS-A and PFS-B in all tested ß-diversity metrics used (Unweighted UniFrac, *p* = 0.001; Weighted UniFrac, *p* = 0.001; Bray–Curtis, *p* = 0.001).

Overall, across the α- and ß-diversity metrics used, PFS group showed a significant reduction of richness, diversity, and composition (low-abundance features), indicating a different gut microbiota structure. Furthermore, the analysis of ß-diversity highlighted two distinct microbial clusters in PFS subjects, with PFS-A more distant to healthy cluster. To gain insight into the microbial differences in PFS patients, we applied multivariate analysis in order to find possible associations between gut microbiota composition, cohort characteristics, and clinical evaluation. No significant association was detected, except the days from suspension of finasteride treatment that seems to be more associated with PFS-A microbiota, even if not statistically significant (*p* = 0.06, data not shown).

### Gut microbiota composition in PFS patients

**Fig. 3:**
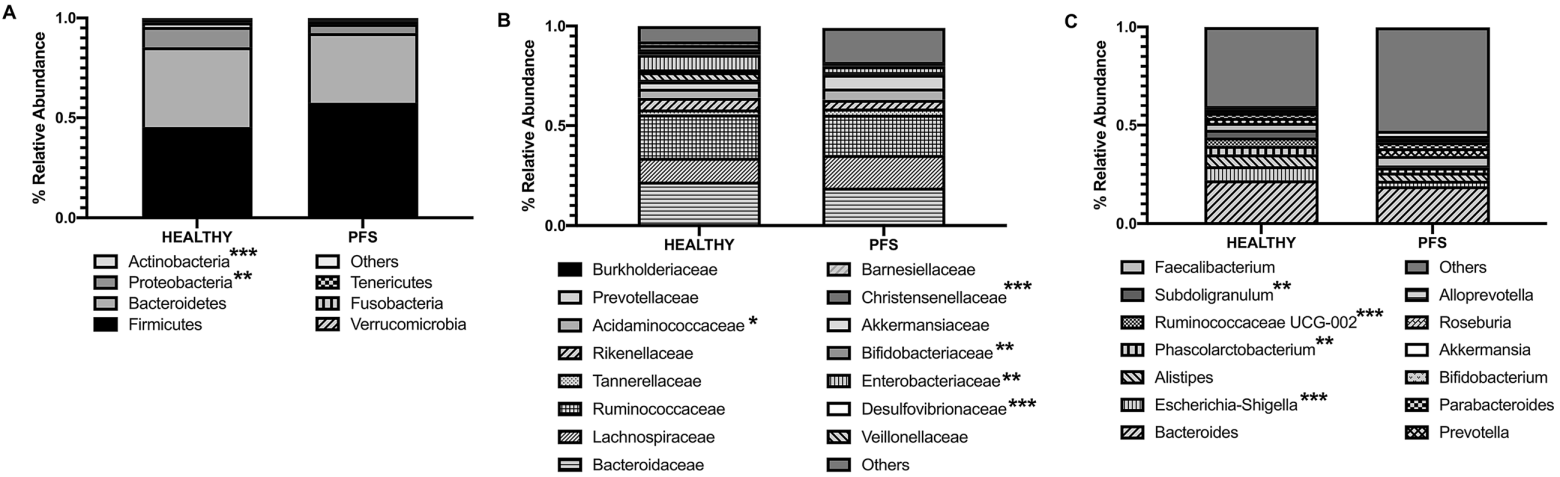
Gut microbiota composition in 10 healthy and 21 PFS patients. Relative abundances are reported at Phylum (**a**), Family (**b**), and Genus (**c**) level. All bacterial taxa present at < 1% relative abundance were grouped into the “Other” classification. *p < 0.05, **p < 0.001, ***p < 0.0001

**Fig. 4:**
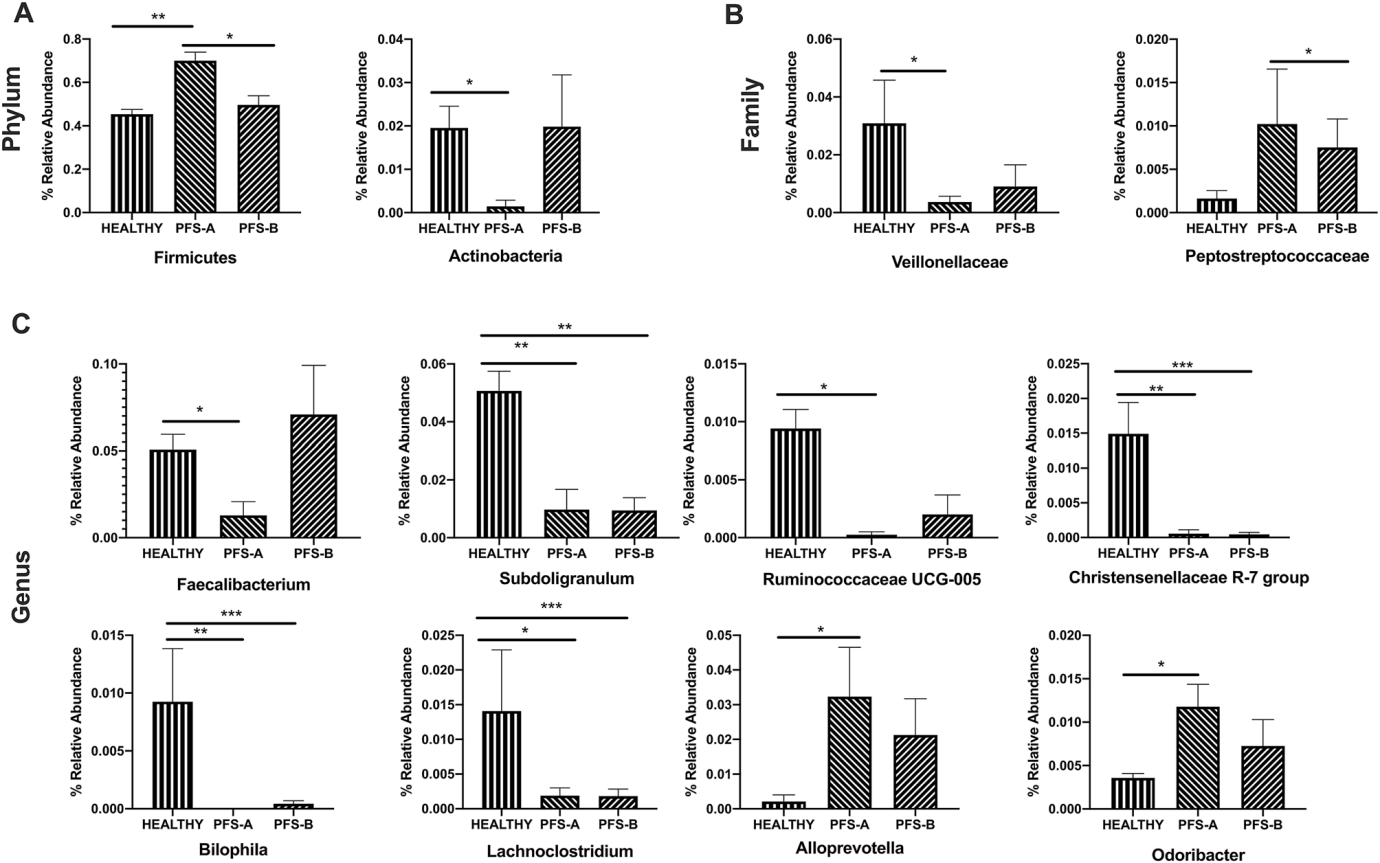
Gut microbiota composition in 10 Healthy and sub-clusters A and B of 21 PFS patients. Relative abundances are reported at Phylum (**a**), Family (**b**), and Genus (**c**) level. Statistical significant values are reported, highlighting the group with greater abundance. *p < 0.05, **p < 0.001, ***p < 0.0001

In addition to the microbiota structure analysis, we also investigated the microbiota composition. As expected, the most relatively abundant phyla in healthy and PFS subjects were *Firmicutes* (mean ± SD; Healthy: 45.4 ± 7.0; PFS: 57.6 ± 17.3; *p* = 0.09) and *Bacteroidetes* (Healthy: 40.4 ± 15.0; PFS: 34.8 ± 17.3; *p* = 0.40) (Fig. 3a). PFS group was significantly depleted in *Proteobacteria* (Healthy: 10.2 ± 15.6; PFS: 4.5 ± 11.3; *p* = 0.009) and *Actinobacteria* (Healthy: 22.6 ± 2.5; PFS: 4.5 ± 3.5; *p* = 0.0003) (Fig. 3a). Among the most relatively abundant families, we found *Acidaminococcaceae* (Healthy: 5.5 ± 10.0; PFS: 4.5 ± 4.8; *p* = 0.04), *Enterobacteriaceae* (Healthy: 7.3 ± 13; PFS: 2.9 ± 11.0; *p* = 0.005), *Bifidobacteriaceae* (Healthy: 15.2 ± 2.2; PFS: 12.3 ± 3.4; *p* = 0.005), *Barnesiellaceae* (Healthy: 1.5 ± 2.8; PFS: 0.3 ± 0.9; *p* = 0.001), *Christensenellaceae* (Healthy: 2.2 ± 2.6; PFS: 0.04 ± 0.1; *p* < 0.001), and *Desulfovibrionaceae* (Healthy: 1.8 ± 1.8; PFS: 0.02 ± 0.01; *p* < 0.001) to be significantly reduced in PFS patients (Fig. 3b). Interestingly, two families closely related to gut microbiota dysbiosis were found different, although not significantly present in PFS subjects in comparison to healthy: *Prevotellaceae* family (Healthy: 3.6 ± 6.0; PFS: 6.9 ± 10.2; *p* = 0.35) and *Akkermansiaceae* (Healthy: 1.1 ± 2.6; PFS: 0.5 ± 1.0; *p* = 0.11) (Fig. 4b). At genus level, the microbiota composition of PFS subjects was characterized by a significant reduction in *Subdoligranulum* (Healthy: 3.8 ± 2.7; PFS: 0.9 ± 1.7; *p* = 0.009), *Phascolarctobacterium* (Healthy: 4.3 ± 4.9; PFS: 2.6 ± 6.7; *p* = 0.009), *Ruminococcaceae* UCG-002 (Healthy: 4.2 ± 3.2; PFS: 0.12 ± 0.27; *p* < 0.0001), and *Escherichia–Shigella* (Healthy: 7.3 ± 13.2; PFS: 2.8 ± 10.8; *p* < 0.001) (Fig. 3c). Furthermore, *Turicibacter* (Healthy: 0.02 ± 0.04; PFS: 0.3 ± 0.5; *p* = 0.16) and *Blautia* (Healthy: 0.2 ± 0.1; PFS: 2.7 ± 4.2; *p* = 0.44), two genera described as related to depressive symptomology, were increased in PFS patients.

To determine whether the two sub-clusters of PFS patients identified have different gut microbiota composition, we analyzed their relative abundances at phylum, family, and genus levels. Sub-cluster A was significantly enriched in *Firmicutes* in comparison to B (*p* = 0.03) and healthy (*p* = 0.002), while *Actinobacteria* was more abundant in healthy than in sub-cluster A (*p* = 0.04) (Fig. 4a). The composition of *Veillonellaceae* family was different between healthy and sub-cluster A (*p* = 0.02), whereas *Peptostreptococcaceae* abundance was higher in sub-cluster A than in B (*p* = 0.03) (Fig. 4b). *Subdoligranulum* spp. (*p* = 0.007, *p* = 0.003), *Lachnoclostridium* spp. (*p* = 0.01, *p* = 0.0006), *Bilophila* spp. (*p* = 0.002, *p* = 0.0002) genera, and *Christensenellaceae* R7 group (*p* = 0.05, *p* = 0.0008) showed a higher abundance in healthy in comparison, respectively, to PFS-A and PFS-B. On the contrary, in comparison to healthy subjects, *Faecalibacterium* spp. (*p* = 0.02) and *Ruminococcaceae* UCG-005 (*p* = 0.02) were decreased only in sub-cluster A, while *Alloprevotella* (*p* = 0.03) and *Odoribacter* spp. (*p* = 0.02) were increased (Fig. 4c).

## Discussion

Data here reported indicate for the first time that gut microbiota composition was altered in PFS patients. In particular, at the phylum level, we here reported a significant increase in *Firmicutes* and a decrease in *Proteobacteria*. Moreover, *Acidaminococcaceae*, *Enterobacteriaceae*, *Bifidobacteriaceae*, *Barnesiellaceae*, *Christensenellaceae,* and *Desulfovibrionaceae* families were significantly decreased. Furthermore, at genus levels *Subdoligranulum*, *Phascolarctobacterium*, *Ruminococcaceae* UCG-002, and *Escherichia–Shigella* were reported to be significantly decreased in PFS patients. Thus, gut microbiota composition is altered in a court of patients who, despite finasteride suspension, report a persistent symptomatology, such as depression, sleep disturbance, and sexual dysfunction [1, 2]. As often observed, depression and insomnia are frequently associated [39, 40]. Indeed, as recently observed, alterations of gut microbiome have been reported in depressive disorders [41–45] as well as in circadian and sleep disturbance [46–48]. In addition, as mentioned above, PFS reported persistent sexual impairment, including feeling a lack of connection between the brain and penis, loss of libido and sex drive, difficulty in achieving an erection, genital numbness, or paresthesia [1, 2]. Indeed, as demonstrated in two different studies, PFS patients showed erectile dysfunction [13, 14]. In agreement with the present results, gut microbiota population is also altered in an experimental model of diabetic erectile dysfunction [49].

Interestingly, under β-diversity analysis, we reported two different sub-clusters of PFS patients significantly different from the healthy group used as control. No statistical differences between the two sub-clusters were observed in terms of age (*p* = 0.5262), BMI (*p* = 0.1927), finasteride treatment period (*p* = 0.817), suspension period (*p* = 0.126), and symptoms reported after finasteride suspension in the questionnaire (data not shown).

At the phylum, family, and genus levels, some gut microbiota populations were modified in the sub-cluster named A but not in B. For instance, at the genus level, *Ruminococcaceae* UCG-005 were significantly decreased in the sub-cluster A of the PFS patients analyzed. It is interesting to note that a similar decrease has been reported by us also in an experimental model of PFS. Indeed, male rats chronically treated with finasteride for 20 days revealed after one month of suspension a decrease of *Ruminococcaceae* [29]. In this context, it is important to highlight, that in agreement with the observation that depressed patients showed a decrease in *Ruminococcaceae* population [33] and *Blautia* spp.[50], PFS patients [13, 14] as well as experimental model of PFS [29] showed depressive symptomatology. Similarly, in agreement with the decrease observed in depressed patients [33, 42, 51], in the sub-cluster A of PFS patients, we also report a decrease in *Veillonellaceae* as well as *Faecalibacterium* spp. population.

The reduction of *Faecalibacterium* spp. has been suggested to be associated with gut dysbiosis and depressive disorders [52]. In addition, this genus is also an important producer of butyrate [53], a short-chain fatty acid which exerts an important role in the communication of gut microbiota and brain [54] and that recently has been proposed to exert a role in sleep modulation [55]. Thus, *Faecalibacterium* spp. might represent a possible target for a therapy aimed to restore gut microbiota alterations in PFS patients and possibly, on the basis of the existence of the gut microbiota-brain axis, also other symptoms observed in these patients. A relationship between gut microbiota population and steroid environment has been also proposed [45, 56, 57]. Indeed, gonadectomy and hormone replacement have a clear effect on gut bacteria in rodents [58–63], for instance, the changes observed in *Ruminococcaceae* after orchidectomy in mice [58]. Alteration in *Ruminococcaceae* also occurred in prostate cancer patients treated with oral androgen receptor axis-targeted therapies [64]. On this basis, it is possible to hypothesize that gut microbiota changes observed in PFS patients may be ascribed to the alteration of steroid environment. Indeed, alterations in the levels of 5α-reduced metabolites of PROG and T have been reported in plasma and cerebrospinal fluid of PFS patients [14, 21, 22]. In addition, since as demonstrated in an experimental model of PFS, alteration of steroid environment also occurs in brain regions [28], the existence of a gut microbiota-brain axis [30–32] may also suggest a role by brain steroids. Finally, a possible contribution by intestinal steroid environment may be also hypothesized. Up to now, steroidogenic capacity in this compartment has not fully clarified. However, microbial species, such as *Clostridium scindens*, have the potentiality to convert glucocorticoids into androgens [65] and therefore may be potential target for the effect of finasteride. In addition, it has been recently demonstrated that type 1 5α-R is expressed in mouse cecum and colon with significant T and DHT levels in these rodent structures as well as in feces of healthy young adult men [66]. Moreover, both in PFS patients as well as in its experimental model [14, 21, 22, 28], finasteride affects the levels of THP, thus a steroid able to interact with GABA-A receptor [24]. In this context, it is important to remember that some members of human microbiota (i.e., *Bifidobacterium* spp. and *Lactobacillus* spp.) encode for genes involved in GABA production, suggesting a microbial participation in the production of this neurotransmitter within the gut [67, 68]. Indeed, GABA-A receptors have been recently identified in the mouse colon [69, 70]. Interestingly, we recently observed that rat colon expresses steroidogenic capability and, in particular, an high production of a T metabolite, such as the 3α-diol [71]. It is important to note that this androgen metabolite, like THP, is able to interact with GABA-A receptors [72] and its levels are affected in PFS patients as well as in its experimental model [14, 21, 22, 28]. This may further support the hypothesis of a role of steroids interacting with GABA-A receptors in the pathogenesis of PFS.

In conclusion, data here reported indicate for the first time that gut microbiota population is altered in PFS patients. With all the limitations represented by the small cohort here considered for this emerging and rare syndrome, these results suggest that gut microbiota composition might represent a diagnostic marker and a possible target for a therapeutic strategy aimed to counteract the important symptomatology occurring in these patients.

## Data Availability

Datasets generated during the current study are available from the corresponding author on reasonable request.
